# Axial Low Back Pain: One Painful Area – Many Perceptions and Mechanisms

**DOI:** 10.1371/journal.pone.0068273

**Published:** 2013-07-02

**Authors:** Matti Förster, Friederike Mahn, Ulrich Gockel, Mathias Brosz, Rainer Freynhagen, Thomas R. Tölle, Ralf Baron

**Affiliations:** 1 Sektion Neurologische Schmerzforschung und–therapie, Klinik für Neurologie Universitätsklinikum Schleswig-Holstein, Campus Kiel, Kiel, Germany; 2 Casquar GmbH, Bochum, Germany; 3 StatConsultGmbH, Magdeburg, Germany; 4 Zentrum für Anästhesiologie, Intensivmedizin, Schmerztherapie und Palliativmedizin, Benedictus Krankenhaus Tutzing, Tutzing, Germany; 5 Klinik für Neurologie, Technische Universität München, München, Germany; The James Cook University Hospital, United Kingdom

## Abstract

Axial low back pain can be considered as a syndrome with both nociceptive and neuropathic pain components (mixed-pain). Especially neuropathic pain comprises a therapeutic challenge in practical experience and may explain why pharmacotherapy in back pain is often disappointing for both the patient and the therapist. This survey uses epidemiological and clinical data on the symptomatology of 1083 patients with axial low back pain from a cross sectional survey (pain*DETECT*). Objectives were (1) to estimate whether neuropathic pain contributes to axial low back pain and if so to what extent. (2) To detect subgroups of patients with typical sensory symptom profiles and to analyse their demographic data and co-morbidities. (3) To compare patients with and without prior intervertebral disc surgery (IVD). Neuropathic pain components could be detected in 12% of the entire cohort. Cluster analyses of these patients revealed five distinct subgroups of patients showing a characteristic sensory profile, i.e. a typical constellation and combination of symptoms. All subgroups occurred in relevant numbers and some showed distinct neuropathic characteristics while others showed nociceptive features. Post-IVD-surgery patients showed a tendency to score more “neuropathic” than patients without surgery (not statistically significant). Axial low back pain has a high prevalence of co-morbidities with implication on therapeutic aspects. From these data it can be concluded that sensory profiles based on descriptor severity may serve as a better predictor for therapy assessment than pain intensity or sole diagnosis alone. Standardized phenotyping of pain symptoms with easy tools may help to develop an individualized therapy leading to a higher success rate in pharmacotherapy of axial low back pain.

## Introduction

Chronic axial low back pain has a high socioeconomic impact. With a lifetime prevalence of 30–50% for moderate and severe chronic back pain in an ageing society it depicts a large clinical and economic burden [1], [2]. In order to develop strategies for pain reduction the underlying pathology needs to be understood more precisely.

Back pain is caused by complex interactions of biological, psychological and social factors. Suffering is often complicated by significantly associated co-morbidities like depression and anxiety. Thus, patient’s quality of life is seriously impaired leading to a complex and demanding therapeutic challenge [3]–[7]. It was assumed that chronic back pain (i.e. pain, which is localized only along the low back for more than 6 months without radicular radiation) represents the prototype of a nociceptive pain state. In chronic nociceptive pain intact nociceptors are activated by tissue damaging stimuli (i.e. ATP, prostaglandins, protons, etc.). This is caused by inflammatory processes in affected muscles, tendons, intervertebral discs and facet joints [8]. In general, pharmacological treatment (e.g. non-steroidal anti-inflammatory drugs, COX-2-inhibitors) will decrease the pain intensity as the underlying pathophysiology is reversible. Neuropathic pain occurs as a result of injured afferent nerves [9]. According to a new concept, neuropathic pain components also contribute to back pain. Thus, back pain can be classified as a mixed pain syndrome in which the overall pain perception of the patients underlies nociceptive and neuropathic pathophysiological mechanisms [10], [11]. The question arises whether mixed pathophysiological mechanisms contribute also to *axial* back pain. This entity has long been considered as purely nociceptive but clinical and diagnostic findings suggest a neuropathic influence [12]. The heterogeneity of different underlying pain mechanisms reveal a much more complex framework than previously assumed and it might explain why pharmacological treatment is often disappointing.

When back pain patients describe their discomfort they frequently use different descriptors for pain qualities and sensory symptoms in various combinations. For nociceptive pain these descriptors include constant aching pain which is located deeply in the back, shooting pain attacks which are often elicited by slight movements or pain which is induced by a slight pressure stimulus at the back. Neuropathic pain components are often associated with burning and tingling sensations [13]. The patient’s experience of pain is interindividually different. However, they use the same descriptors for pain states that could mechanistically be added to either neuropathic or nociceptive conditions when asked to describe their pain symptoms. [14]. It has been suggested that a symptom constellation (profile) allows better approximation to the underlying pathophysiological process in the afferent system than definite single sensory symptoms [14]–[16]. The pain*DETECT* questionnaire (PD-Q) was designed to screen for neuropathic pain on such considerations. It allows discrimination between neuropathic and nociceptive pain components in chronic pain syndromes through a score system based on 9 questions (7 pain descriptor questions and two concerning radiation and pain course). A validation study was performed in back pain patients [17]. Here, it was found that 37% of an unselected low back pain cohort (n = 7772) showed a predominant neuropathic pain component. This subgroup suffered from higher pain intensities, too. The pain descriptor questions from the PD-Q can be used to create symptom profiles via statistical cluster analyses that are indicative of neuropathic or nociceptive pain [17]. A precise assessment of the somatosensory profile in back pain patients may help to understand the contribution of nociceptive and neuropathic pain components to the overall back pain. Additionally, co-morbidities (e.g. depression, sleep disturbances) have a higher prevalence in neuropathic pain syndromes compared to a matched population [7], [18]. Previous analyses of low back pain pain*DETECT* data revealed a higher prevalence of depression, panic and anxiety disorders and sleep disorders [17], [19]. Also, patients with radiculopathy showed similar frequencies of co-morbidities as classical neuropathic pain syndromes [20]. Subsequently the patientś description of symptoms might be used to develop a personalized and mechanism-oriented treatment concept for back pain patients in the future [8], [21], [22].

We analysed epidemiological and clinical data of 1083 patients with axial low back pain from a cross sectional cohort survey in Germany (pain*DETECT*) performed in collaboration with the German Research Network on Neuropathic Pain (DFNS). The following hypotheses were tested:

1. Neuropathic pain contributes to the overall pain experience in axial low back pain.
2. Subgroups with typical sensory symptom profiles that are indicative of neuropathic or nociceptive pain exist and show characteristic demographic data and co-morbidities.
3. Intervertebral disc surgery has an impact on neuropathic pain components.

## Materials and Methods

### Ethics Statement

All data was analysed anonymously after patient’s informed consent.

### Study Population

The investigation was performed as a non-interventional study at 450 outpatient centres in Germany (general practitioners, rheumatologists, orthopaedists and pain specialists) from January 2006 to December 2010. Patients with lumbar axial back pain, at least 18 years old who had previously given written consent, used a hand-held computer (Palm Tungsten E operating on OS5.4) to complete electronic questionnaires for the epidemiological and clinical survey [23]. At intervals data transfer performed under secure conditions, with anonymisation and encryption to a central pool data base were done. Physicians did not receive a financial incentive. The study protocol was approved by the ethical committee of the University of Düsseldorf.

The patient selection was done based on pain drawings performed by the patients in the palm top device. This device is equipped with a body drawing with 34 predefined body areas. The patients were asked to mark their body areas with the most prominent pain. Only back pain patients in whom the lumbar axial back was the predominant complaint were included in the study. Patients with pain radiating into the leg or any other body site were excluded to ensure a homogenous group.

### Data Collection

To assess the somatosensory symptoms within the painful lumbar area the pain*DETECT* questionnaire (PD-Q) was used. The questionnaire was originally developed to identify neuropathic pain components and was validated in a cohort of patients that included lumbar back pain [17].The patients could rate the perceived severity of each symptom from 0–5 (never, hardly noticed, slightly, moderately, strongly, very strongly). In detail seven questions address the following sensory symptoms: question 1 - spontaneous burning pain, question 2– spontaneous prickling sensations, question 3– pain evoked by light touch (allodynia), question 4– spontaneous pain attacks, question 5– pain evoked by thermal stimuli, question 6– numbness, question 7– pressure pain. Additionally, patients had to describe the pain course (options: persistent pain with fluctuations, persistent pain with pain attacks, pain attacks with persistent pain, pain attack with free intervals). A PD-Q score was calculated by adding the score values of the seven questions and the values assigned to each course possibility. A total score of 38 could be reached. Cut-offs were >18 for a >90% probability of neuropathic pain components (i.e. positive) and <13 for nociceptive components (i.e. <15% probability of neuropathic components, negative). Score values in between these two were considered as unclear, i.e. a neuropathic component can be present. Sensitivity and specificity for this screening test are both 84% with a positive predictive value of 83%. Test-retest-reliability for the PD-Q in a back pain cohort shows good reliability (unpublished data; data will be presented as poster at NeuPSIG, Toronto, 2013; authors: R. Baron, R. Freynhagen, U. Gockel, T. Kohlmann, T. Keller, E. Stemmler and T. R. Tölle).

Pain intensity was acquired on a visual analogue scale (VAS) for worst and average pain in the past four weeks as well as current pain.

Standard demographic questions and the following questionnaires were used to assess co-morbidities: for sleep disturbances the Medical Outcomes Study Sleep Scale (MOS-SS) and for depressive, panic and anxiety disorders the German-language Patient Health Questionnaire (PHQ-D, short form) [24], [25].

### Statistics

Descriptive statistical analyses were performed with the SAS package, version 9.2. Relations between two dichotomous variables were assessed by 2×2 contingency tables, relations between categorical data in general using k×m contingency tables. Continuous variables were presented within tables by mean plus/minus standard deviation. Categorical data were tabulated using frequencies and percentages.

In order to identify relevant subgroups of patients who are characterized by a typical constellation of 7 sensory symptoms cluster analyses were performed (PROC FASTCLUS) as described before [18]. The clustering bases on Euclidian distances.

Two calculations were performed: (1) The absolute values for each symptom intensity score were assessed. (2) To eliminate inter-individual differences of the general perception of sensory stimuli (differences in individual pain perception thresholds) the intensity scores of the questions were re-calculated. In detail, the given 0–5 score of each question was subtracted by the mean of all values marked in the 7 questions. In this individual score values above 0 indicate a sensation which is more intensive than the individual mean pain perception, values below 0 indicate a sensation which is less intensive than the individual mean pain perception.

We identified a 5-cluster solution to be the optimal compromise between group size and stability of the clusters. The clusters are represented by the patterns of questionnaire scores, thus showing the typical pathological structure of the respective group. As this is a heuristic approach no statistical analysis was performed.

Co-morbidities were analysed with Tukey’s studentized range HSD test.

## Results

### Epidemiological Features, Pain Intensity, and Sensory Symptoms

1083 patients (453 male, 630 female; age range 58±15 years) fulfilled the selection criteria as described above. The demographic profiles of the patients are shown in Table 1. 158 patients (14.5%) had undergone IVD-surgery prior to the investigation. The VAS intensity values for “worst pain”, “average pain” and “current pain” were 7.2, 5.3 and 4.7, respectively (Table 2).

**Table 1:** Demographic data and co-morbidities of 1083 patients.

Included patients (n)	1083	% 100
Male	453	41.8
Female	630	58.2
Age (years)*	58.0±15.0	
Male*	55.8±14.6	
Female*	59.5±15.0	
**Height (cm)**		
Male*	177.5±7.7	
Female*	164.2±7.1	
**Weight (kg)**		
Male*	86.2±16.6	
Female*	74.3±15.8	
**BMI (kg/m^2^)**		
Male*	27.4±4.8	
Female*	27.6±5.6	
**PD-Q (n)**		
Negative	750	69.25
Unclear	202	18.65
Positive	131	12.10
**IVD-surgery**	158	14.5
**Depression (PHQ-9 values)**		
None (0–4)	311	28.7
Mild (5–9)	406	37.5
Moderate (10–19)	331	30.6
Severe (20–27)	35	3.2
**Panic/anxiety disorder**	47	4.3
**MOS-SS**		
Sleep disturbance		40.3
Optimal sleep		43.9
Somnolence		37.3
Sleep quantity (hours)	6.1	
Sleep adequacy		51.3

BMI: Body mass index; PD-Q: painDETECT questionnaire; IVD: intervertebral disc; PHQ-9: nine item scale of Patient Health Questionnaire; MOS-SS: Medical Outcome Study sleep scale; * mean ± standard deviation.

**Table 2:** Pain and perceived sensory symptoms in patients with axial low back pain.

	total	Cluster 1	Cluster 2	Cluster 3	Cluster 4	Cluster 5
n	1083	237	229	162	175	280
VAS (worst)*	7.2±2.2	7.6±2.2	7.1±2.2	6.9±2.3	7.7±1.9	6.7±2.3
VAS (average)*	5.4±2.2	5.3±2.3	5.3±2.2	5.5±2.2	5.9±1.9	4.9±2.3
VAS (current)*	4.7±2.6	4.6±2.7	4.7±2.5	5.1±2.4	5.4±2.5	4.3±2.7
**Clinical relevant complaint (%)** **						
Burning	16.2	1.7	1.3	25.9	56.6	9.6
Prickling	10.9	2.5	3.1	36.4	11.4	9.3
Allodynia	5.6	0.4	7.9	3.1	8.6	7.9
Attacks	27.0	75.1	3.9	21.0	27.4	8.2
Thermal	5.6	3.4	3.9	2.5	1.1	13.6
Numbness	4.9	0.8	1.3	21.0	0.0	5.0
Pressure	22.8	20.7	42.8	8.6	33.7	9.6

* mean ± standard deviation:

** score >3 (strongly, very strongly).

Table 2 illustrates the quality and intensity of specific sensory symptoms that were regarded as clinically relevant in the entire population (column “total”). A symptom was considered clinically relevant if the patient marked a score of >3 (strongly or very strongly). The most prominent symptoms were pain attacks and pressure induced pain described as clinically relevant in 27% and 22.8%. Clinically relevant touch evoked allodynia (5.6%) and thermal induced pain (5.6%) as well as numbness (4.9%) were uncommon symptoms.

**Figure 1:**
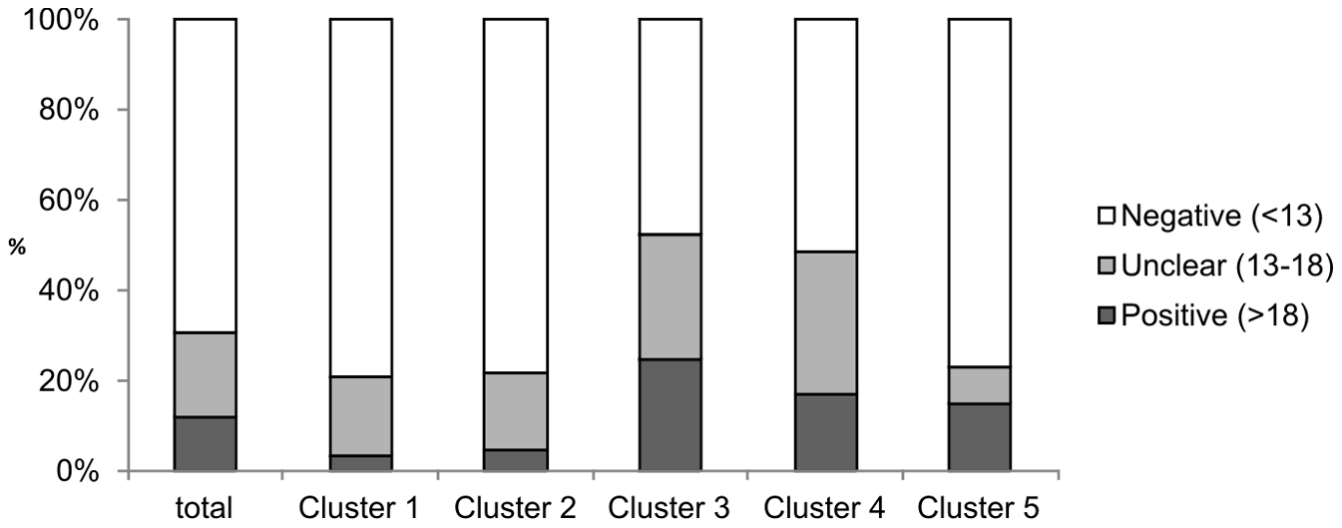
Differences in PD-Q scores in the subgroups. The different scores calculated from the PD-Q are shown, revealing the proportion of positive, i.e. neuropathic and negative, i.e. non-neuropathic as well as unclear results. Patients from clusters 3 and 4 showed the tendency to score more neuropathic than those from clusters 1, 2 and 5.

Of all patients 12.1% scored positive on the PD-Q (i.e. neuropathic elements likely, n = 131), while 69.3% scored negative (i.e. neuropathic elements unlikely, n = 750) and 18.7% unclear (n = 202) (Table 1, figure 1 “total”).

### Subgroups of Patients Based on Sensory Abnormalities

**Figure 2:**
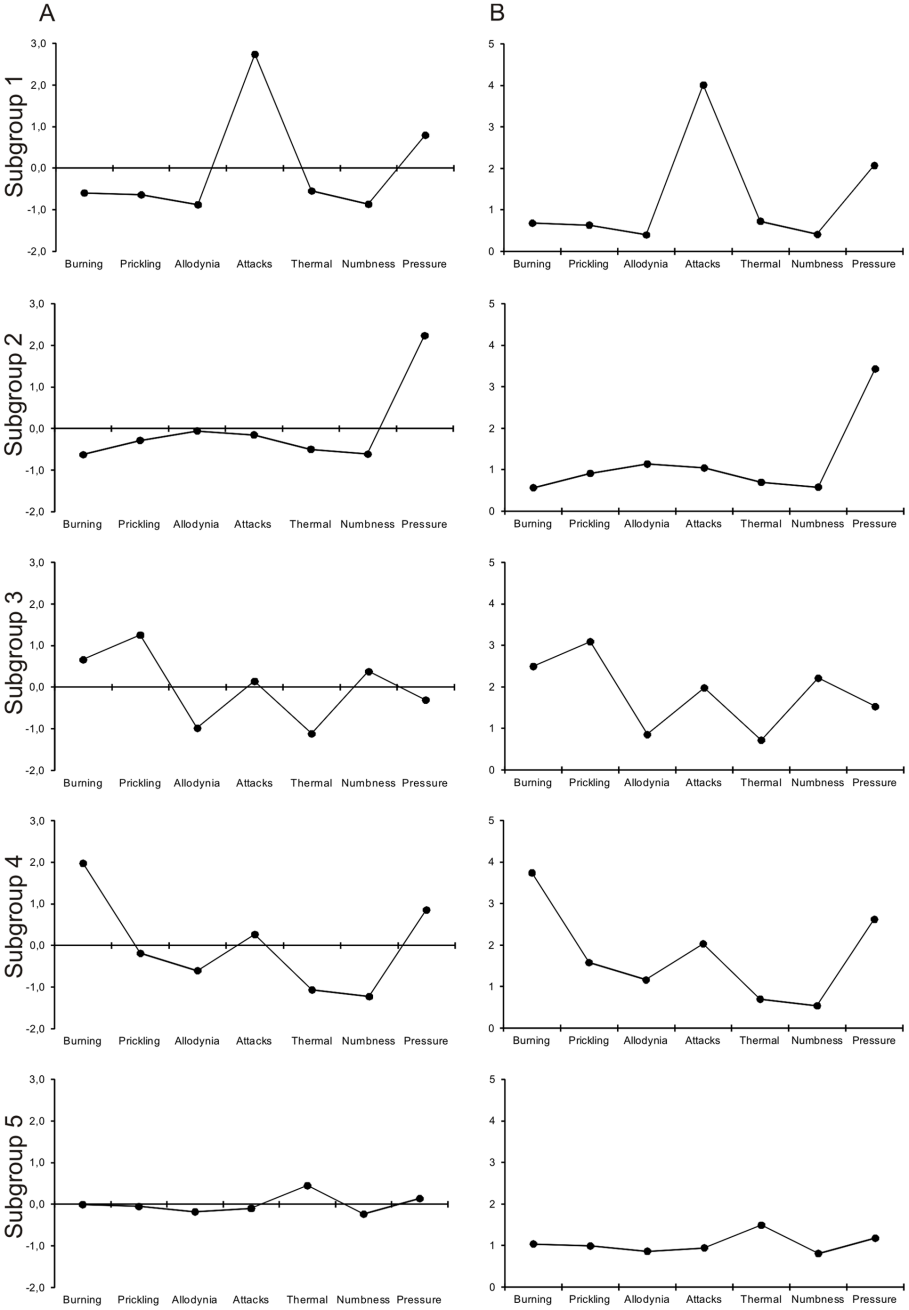
Subgroups of patients based on their sensory symptoms. To identify relevant subgroups of patients who are characterized by a characteristic symptom constellation a hierarchical cluster analysis was performed. The clusters are represented by the patterns of questionnaire scores (A: adjusted individual mean; B: non-adjusted values), thus showing the typical pathological structure of the respecting group. By using this approach five clusters with distinct symptom profiles could be detected in the cohort. Sensory profiles show remarkable differences in the expression of the symptoms. Subgroup 5 does not show any outstanding symptoms and low prevalence of symptoms in general.

A cluster analysis was performed to identify relevant subgroups which present with a characteristic constellation of sensory symptoms. Figure 2A shows the different clusters with distinct symptom profiles and table 2 their corresponding frequencies. In the five-cluster-solution we found sensory profiles with remarkable differences in the expression of the experienced symptoms. All subgroups represented a relevant part of the cohort (14–26%).

Cluster 1 (n = 237, 21%) and cluster 2 (n = 229, 21%) demonstrate only one dominating symptom, i.e. painful attacks or pressure induced pain, respectively. In cluster 4 (n = 175, 16%) pressure-induced pain and burning sensations were prominent whereas nearly all other symptoms were moderately expressed. Cluster 3 (n = 162, 14%) is characterized by relevant prickling and burning sensations. The profile of cluster 5 (n = 280, 26%) is mainly concentrated around the zero-line for all parameters. This indicates that the patients tend to mark a similar score for all questions. Although the average pain intensity was VAS 4.9 in this group all sensory symptoms were only rated in the range of “never” to “hardly noticed” (see non-adjusted profile, figure 2B).

PD-Q-score “positive” was found with the highest frequency in clusters 3 and 4, while clusters 1 and 2 scored significantly lower (24.7% and 17.14% in clusters 3 and 4, respectively, 3.4% and 4.8% in clusters 1 and 2, respectively; see figure 1). Patients from cluster 4 had the highest values of spontaneous pain, while those from cluster 5 had the lowest values.

### Co-morbidities

All patients were screened for severity of depression and panic/anxiety disorders as well as noticeable problems in their sleep behaviour. These co-morbidity data are depicted in table 1. Additionally, descriptive analysis on co-morbidities between the clusters was performed. The severity and frequencies of the investigated disorders are shown in table 3. Statistical significance was achieved between clusters 5 and 2 and 4 for sleep disturbance, between 5 and 4 for somnolence, between 5 and 2 and 3 for sleep quantity and between 5 and 2 for sleep adequacy (for all of the above: Tukey’s studentized range HSD test p<0.05). From these data it can be concluded that subgroup 5 is affected by co-morbidities to the smallest extent of all groups that were analysed.

**Table 3:** Distribution of co-morbidities within symptom-clusters.

	Cluster 1	Cluster 2	Cluster 3	Cluster 4	Cluster 5
N	237	229	162	175	280
**Depression (PHQ-9 values)**					
Mild (5–9)	42.6	31.4	37.6	39.5	36.8
Moderate (10–19)	27.9	33.2	35.8	33.1	26.1
Severe (20–27)	3.8	3.5	3.1	4.0	2.1
**Panic/anxiety disorder**	5.1	3.1	5.6	3.4	4.6
**MOS-SS**					
Sleep disturbance	40.8	42.7	41.5	42.8	35.6
Optimal sleep	47.7	38.4	42.6	42.9	46.4
Somnolence	36.6	38.8	38.1	40.6	34.1
Sleep quantity (hours)	6.4	6.2	6.2	6.4	6.6
Sleep adequacy	54.4	50.4	54.2	53.0	60.1

### IVD-surgery

**Figure 3:**
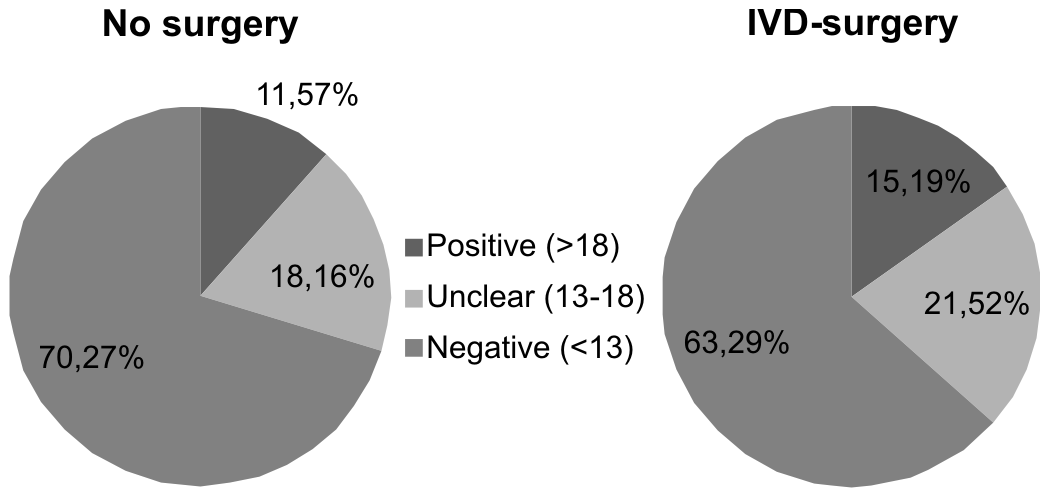
Differences in PD-Q scores after IVD-surgery. The pie-chart depicts the proportion of patients with and without IVD-surgery scoring “positive”, “unclear” or “negative” in the PD-Q. There are no significant differences between the respective groups (χ^2^-Test, p = 0.2215).

Of the patients with axial low back pain without IVD-surgery 70.3% scored negative in the PD-Q (n = 650), while 11.6% scored positive (n = 107). Post-IVD-surgery patients were negative in 63.3% (n = 100) and positive in 15.2% (n = 24, Figure 3). The frequency of score values between the surgery and non-surgery groups failed to be significant (χ^2^-Test, p = 0.2215). An analysis of the different clusters was not performed because of low patient numbers within the corresponding subgroups.

## Discussion

The study revealed three main findings:

1. Neuropathic pain components are prevalent in axial low back pain patients in more than 10% and co-morbidities occur to a large extent.
2. Patients with chronic axial low back pain can be subdivided into subgroups with distinct patterns of perceived sensory abnormalities (sensory profiles).
3. IVD-surgery influences the pain experience towards a more neuropathic perception.

### Neuropathic Pain and Constellation of Sensory Symptoms

In this study 12.1% of axial low back pain patients scored positive on the PD-Q, i.e. suffered from sensory symptoms which are indicative of neuropathic pain components [17]. While others have found a higher proportion (36–55%) of neuropathic pain in back pain cohorts [1], [2], [11], [17] our finding matches studies that have been published previously [19]. Higher prevalence can be accounted by an overrepresentation of neuropathic pain patients in specialist centers comparable to the above mentioned studies [26].

Our study revealed that patients with axial lumbar back pain are characterized by a variety of different pain types and sensory symptoms that are mechanistically distinct. We performed a cluster analysis to identify relevant subgroups of patients who demonstrate characteristic sensory profiles (Fig. 3). In order to tailor an individual therapeutic concept relying on symptom assessment the underlying pain-generating pathological mechanisms need to be elucidated [8], [21], [22].

Nociceptive back pain is evoked by noxious stimulation of deep somatic structures in the lumbar spine, often induced by ingrowth of small nociceptive nerve-fibers into degenerated intervertebral discs. It is characterized by a dull and aching quality localized in the back [11], [27]. Furthermore, due to the musculoskeletal nature of the pain the muscle is explicitly tender to pressure stimuli [28]. These mechanisms are ideally mirrored by cluster 2 which is dominated by pressure induced pain. Thus, it is likely that these patients suffer of nociceptive pain (pain*DETECT* positive: 4.8%).

Patients who fall into subgroup 1 (22%) predominantly suffer from “pain attacks” (pain*DETECT* positive: 3.38%). They express that even the slightest movement of the affected lumbar spine is capable of inducing a very severe, short lasting pain in the back that ceases immediately after seconds. However, in contrast to radicular pain, it is located in the lumbar region. Physiologically, it can be assumed that these attacks are evoked by ectopic discharges emanating from sensitized nerves e.g. innervating facet joints and outer layers of intervertebral discs [12]. Secretion of pro-inflammatory cytokines and neurotrophins as response to constant pressure in the vicinity of the affected nerve seem to be the critical underlying pathophysiological process [12], [29].The effect of cyclic mechanical stress on the production of inflammatory agents may induce a synergistic effect of simultaneous mechanical and chemical irritation of the annulus fibrosus cells on the reactionary production of pain mediators (PGE2) [30].

Subgroups 3 and 4 (together 31% of the entire cohort) are characterized by burning and prickling sensations (pain*DETECT* positive: 25% (cluster 3) and 17.2% (cluster 4)). These symptoms are characteristic for neuropathic pain syndromes [13]. Accordingly these clusters may represent the neuropathic subgroups in axial low back pain. Pathophysiological concepts describe an isochronic occurence of neuropathic and nociceptive components in axial back pain [10]. Normally, intervertebral discs are only sparsely innervated; afferent fibers are exclusively located at the outer layer of the annulus fibrosus [12]. This situation changes dramatically if the disc tissue is damaged. Diseased human discs are heavily invaded by blood vessels and small nociceptive nerve-fibers [31]. Macrophages secrete pro-inflammatory cytokines; in particular TNF-α and other neurotrophins act as growth factors [29]. Thus, nociceptive fibers start sprouting from the outer part into the inner areas of the disc including the nucleous pulposus. One could hypothesize, that besides nociceptive mechanisms continuous compression of axonal sprouts within diseased discs suffer damage due to compressing forces. As a consequence these damaged afferent fibers in the disc give rise to neuropathic pain mechanisms represented by specific symptoms [8].

Interestingly, patients in subgroup 5 did not indicate distinct sensory abnormalities and scored very low sensory symptom severity despite the fact that the average spontaneous pain intensity was VAS 4.9. Sensory symptoms do not seem to be of clinical importance to the patients in subgroup 5 even though they reach a positive score on the pain*DETECT* in 15%. This reveals, that a group of patients with clinically significant pain intensity exists whose pain experience is not adequately covered by the questions of the PD-Q.

In conclusion, besides nociceptive pain mechanisms neuropathic components also play a key role in the pathophysiology of axial low back pain. Obviously, these mechanisms play in concert so that the investigating physician faces a mixed pain syndrome. The analysis of the different pain components may provide a basis to the most promising therapy.

### Co-morbidities

Back pain patients show a high frequency of co-morbidities such as sleep disorders, depression and panic/anxiety disorders [17]. More specifically in patients with neuropathic back pain these disorders occur quite often [19], [20]. Our data supports this finding, as a large group of the patients showed pathological sleeping behaviour and signs of depression or panic/anxiety. However, compared to large epidemiological studies on unselected back pain and radiculopathy patients or classical neuropathic pain syndromes (e.g. diabetic polyneuropathy) the axial low back pain cohort in this study complained to a lesser extent of these co-morbidities [17], [18], [20].

Between the clusters a consistent distribution of co-morbidities was not prevalent. It is notable that patients from cluster 5 experienced an almost normal sleep adequacy with close-to-normal values for sleep disturbance and somnolence. Besides, 35% of these patients did not reveal signs of depression, while only 2.1% suffered from a severe depression (see table 3). This is notable, because 15% score positive on the PD-Q while showing a sensory profile without discrimination between different items.

Thus, treatment response differences between axial low back pain patients and other neuropathic pain syndromes may not solely be explained by differences in the prevalence of co-morbidities.

### Impact of IVD-surgery on Neuropathic Back Pain

The PD-Q score was higher in patients who underwent surgical interventions prior to our study. Although this analysis was underpowered and did not reach a statistically significant level, this finding could depict a shift to neuropathic pain components. Damage caused by surgical interventions (e.g. due to mechanical, thermal and chemical stimuli) to surrounding tissues including nerve fibers could explain this observation. High-risk surgical techniques giving rise to chronic postoperative pain have been identified [32]. Back surgery in particular leads to severe tissue destruction [33], [34]. Direct damage, inflammatory processes and chronic pressure interfere with physiological neuronal function and may lead to the rise of neuropathic pain. However, larger studies need to be conducted in order to support this theory.

### Limitations

In this cross-sectional survey patients filled out several self-assessed questionnaires (PD-Q, MOS-SS, PHQ-D). These tools are limited by the comprehension of the questions (e.g. does the patient understand what is intended by the question “does your skin feel numb?”). However, the large cohort of 1083 selected patients from 450 centers is expected to rule out inaccuracies. Also, sensory symptoms and co-morbidities are not the only variables which determine the response to analgesic treatments. The pharmacological response is also influenced by genetic susceptibility and psychological factors such as catastrophizing and expectation which were not assessed in the present investigations.

Another methodological consideration may limit the results of our study and questionnaire-based studies in general: Despite good sensitivity and specificity of the PD-Q [17], the question remains whether the distinction between neuropathic and nociceptive symptom profiles truly represents the biological background of pain or whether it may be an artificial effect. Neuropathic pain has to be considered as a syndrome consisting of a constellation of symptoms and signs. Its cause may by distinct but most often relying on multiple mechanisms. A grading system was introduced in 2008 by Treede et al. due to the lack of a diagnostic tool [35]. Thus, the lack of a gold standard leaves a degree of uncertainty of the calculated sensitivity and specificity values of the PD-Q [36]. However, quantitative sensory testing profiles reflecting somatosensory abnormalities separated well within the categories of the clinical grading system [37]. Despite these limitations, other questionnaires were able to show distinct symptom profiles that distinguish between neuropathic and nociceptive pain patients [14], [38]. A more sophisticated approach was suggested by a group that linked questionnaires with somatosensory testings to better understand mechanisms of neuropathic pain [39]. However, it is important that future work validates the existence of a questionnaire-based profile distinction.

### Conclusion

Our data suggest that sensory profiles based on descriptor severity may be a better predictor for therapy assessment than pain intensity alone especially considering the various underlying mechanisms operating in concert. Phenotypic differences in sensory profiles and co-morbidities as shown in this study as well as in others might explain some of the variance in treatment response and help to tailor an individualized therapy for patients in the future. To achieve this ultimate goal a phenotype-pathophysiology-dependent adaption of the therapeutic regimen for individual patients is required for a more satisfying rate of therapy responders.
